# Biofilm Composition Changes During Orthodontic Clear Aligners Compared to Multibracket Appliances: A Systematic Review

**DOI:** 10.3390/microorganisms13051039

**Published:** 2025-04-30

**Authors:** Alba Belanche Monterde, Javier Flores-Fraile, Esteban Pérez Pevida, Álvaro Zubizarreta-Macho

**Affiliations:** 1 Department of Surgery, Faculty of Medicine and Dentistry, University of Salamanca, 37008 Salamanca, Spain; belanche.alba@usal.es (A.B.M.); eperezperida@usal.es (E.P.P.); alvaro.zubizarreta@usal.es (Á.Z.-M.); 2 Department of Implant Surgery, Faculty of Health Sciences, Alfonso X el Sabio University, 28691 Madrid, Spain

**Keywords:** brackets, clear aligners, biofilm, microbiome, periodontitis, bacteria

## Abstract

Clear aligner treatment seems to be a good option for the periodontal patient by the reason of being removable. Multibracket appliances are more difficult to mantain clean and some bacteria might prefer to adhere on the archwire. A systematic review was carried out using 4 electronic databases (Pubmed-Medline, Scopus, Cochrane and Web of Science). The selected trials included quantitative (Shannon index, Simpson index, relative abundances) and/or qualitative (alpha and beta diversity) analysis in patients using clear aligners and multibracket appliances. Initially, a total of 123 articles were found after selecting clinical trials. The inclusion and exclusion criteria were applied by two authors. Finally, 20 articles were selected for the systematic review. The results showed that clear aligner treatment produced less dysbiosis in the selected bacteria compared to multibracket appliances. However, some microbiological changes were observed in some articles during clear aligner use. Oral dysibiosis was related with intestinal dysbiosis, inflammatory response and even cancer. The Firmicutes/Bacteroidetes ratio showed to have a very important role in this development. Periodontitis is also a bacterial disease and clear aligners were recommended to periodontal risk patients. Clear aligner treatment obtained less supra and subgingival biofilm changes compared with multibracket appliances but some bacteria were altered during treatment.

## 1. Introduction

Recently, the prescription of clear aligners has increased due to the aesthetic advantages this orthodontic system allows. This technique was established based on the design of a positioner that Keslign created. It requires the manufacturing of individual splints for consecutive set-ups which simulate the sequenced dental movements [1]. Aligners have been recommended to high-periodontal-risk patients due to the better periodontal response they might obtain compared to fixed appliances [2]. It has been observed that the oral biofilm is altered with orthodontic treatment. The long-term plaque biofilm observed during orthodontics is mostly composed of anaerobic and cariogenic bacteria such as Streptococcus Mutans (SM). In addition, it has been observed that periodontal bacteria such as *Aggregatibacter Actinomycetemcomitams* are less predominant during orthodontic treatments. However, there could be differences between fixed appliances and clear aligners because there are bacteria that have more affinity with metallic surfaces than plastics [3]. Previous systematic reviews with the same aim showed that clear aligners have a good effect on the oral microbiome compared to braces. Clear aligners were shown to better maintain the balance between microorganisms in the oral cavity; however, there are few articles focusing on this [4]. During the orthodontic movement, a periodontal ligament hypoxia is produced. Interleukin-6, tumor necrosis factor-alpha, and another cytokines activate the osteoclasts leading to bone resorption in the pressure zones, allowing for the movement of teeth. In the aligner treatment, the application of the forces is not continuous, while this force is continuous in when braces are used as the treatment due to their elastic archwire. This fact also seems to be important to gingiva health [5]. On the other hand, in periodontitis progression, a pathological inflammatory process occurs, leading to the continuous destruction of the ligament and bone support. Although periodontitis is a disease with a multifactorial etiology, insufficient oral hygiene has been highlighted as the main risk factor. Poor oral hygiene produces some changes in the composition and conformation of the oral biofilm. In addition, orthodontic treatment can benefit the periodontal ligament. The balanced occlusal contacts obtained via orthodontics improve prognosis regarding periodontitis and increase the durability of the teeth [6,7]. Moreover, periodontal bacteria are identified that belong to the red and orange complexes. The red complex includes *Porphyromonas gingivalis*, *Tannerella forsythia,* and *Treponema denticola*. Subgingival microbiota were previously used to study the oral biofilm in the periodontal pockets. Also, saliva is used to evaluate oral biofilm alterations. Saliva samples are easier to collect and the methodology is less invasive. Some bacteria are usually detected in subgingival sulcus plaque and saliva; for example, *Porphyromonas gingivalis* and *Treponema denticola*. Polymerase chain reaction (PCR) has been used in in vivo trials in order to identify the ribonucleic acid (RNA) of the periodontopathogens [8]. The quantity and quality of bacteria are also influenced by genetics, diet, and socioeconomic and racial factors. In addition, systemic diseases such as diabetes mellitus are related to oral dysbiosis, so it is important to take this into account during orthodontics. Clorhexidine is effective in controlling oral infections and the stability of the biofilm in patients with systemic disorders [9,10]. However, currently, the use of mouthwashes containing clorhexidine and silver nanoparticles by orthodontic patients show limited efficacy in the control of the Gingival Index, Plaque Index, Pocket Probing Depth, and Streptococcus Mutans (SM) levels [11]. Toothpastes containing clorhexidine and amine fluoride are also used to reduce the risk of periodontitis and gingivitis during orthodontics [12].

The purpose of this systematic review was to evaluate the variations in the oral microbiota that occur during orthodontic treatment with clear aligners and compare them with those observed during the use of multibracket appliances.

## 2. Methodology

### 2.1. Search Strategy

The electronic search was conducted through four databases (PubMed Medline, Cochrane, Scopus and Web of Sciences) using the following PICO question: “Do clear aligners modify the oral biofilm in a different manner than bracket appliances?” The systematic review was registered in INPLASY with the number INPLASY202540079. No manual search was performed, and no year restrictions applied. In addition, clinical trials were selected in the databases. The systematic search of trials started in March 2022 and ended in February 2025. The strategy search “((biofilm) OR (bacteria) OR (microbiota) AND (clear aligners) AND ((brackets) OR (orthodontics))” was used to find the clinical trials. The PRISMA checklist was followed when conducting the systematic review. The study population was patients treated with clear aligners. Patients were without any contraindications that could modify the oral biofilm, such as smoking habits or medicine intake. Only patients with good periodontal status were included (no radiographic signs of bone loss or periodontal inflammation, no pocket depth more than 3 mm, no teeth mobility, no gingiva bleeding, and good hygiene status). No restrictions regarding the follow-up were applied. The interventions focused on microbiological changes before and after a period of orthodontic treatment, measured via subgingival and/or supragingival bacterial analysis. Multibracket orthodontics and clear aligners were compared before and after treatment. Outcomes were assessed based on the type and quantity of the bacteria observed before and after treatment. Through this systematic review, the authors aimed to evaluate whether the proportion of periodontopathogenic bacteria increased with clear aligner treatment.

### 2.2. Inclusion and Exclusion Criteria

The systematic review was conducted taking into account the inclusion and exclusion criteria, which were selected prior to the review by two authors (A.B.M and E.P.P). Initially, the main idea of the study was to perform a meta-analysis; however, most of the articles were in vivo trials and analyzed qualitative variables. Therefore, a systematic review was conducted, and the inclusion and exclusion criteria were reestablished. The inclusion criteria were in vivo and in vitro trials, observational and experimental trials, in vitro trials with clear aligners, and trials that evaluated the oral microbiome during aligner treatment. Articles that showed changes in oral biofilm obtaine with multi-bracket appliances versus clear aligners were also included. As exclusion criteria, we ruled out systematic reviews and meta-analyses focusing on clear aligners, trials that used fluoride or remineralizing coating agents in clear aligners, and trials focusing on clear aligners that did not evaluate the biofilm. Pregnant patients, children, patients with smoking or alcohol habits, and diabetic patients were excluded from the review. No year or language restrictions were applied. There were no restrictions regarding prior periodontal treatment in these patients, and the articles did not address the decision to perform orthodontic treatment in periodontal patients.

### 2.3. PICO Question

- Population (P): patients using clear aligners as orthodontic treatment.
- Intervention (I): microbiological analysis of the oral biofilm obtained from subgingival or supragingival saliva. The most common methodology used to assess biofilm in the included studies was polymerase chain reaction (PCR) and 16-gene rRNA sequencing in private micro-biology laboratories.
- Comparison (C): group with brackets (self-ligating, lingual, and conventional buccal brackets).
- Outcomes: differences between fixed brackets and clear aligners in the oral microbiota that could affect periodontal status.

### 2.4. Risk of Biases

Risk of bias was assessed separately by two authors (A.B.M. and E.P.P.). Opinions were then pooled, and in cases of disagreement, a third author (J.F-F.) made the decision. Risk of bias was assessed using the JADAD scale.

### 2.5. Data Extraction

Two authors (A.B.M. and J.F-F.) extracted data on the proportions and bacterial spe-cies found in each clinical trial and recorded them in Excel spreadsheets. All authors ana-lyzed the data to assess trends in biofilm changes and correctly identify periodontopatho-genic bacterial groups and the Firmicutes and Bacteroidetes families.

## 3. Results

### 3.1. Analysis of Results

**Figure 1:**
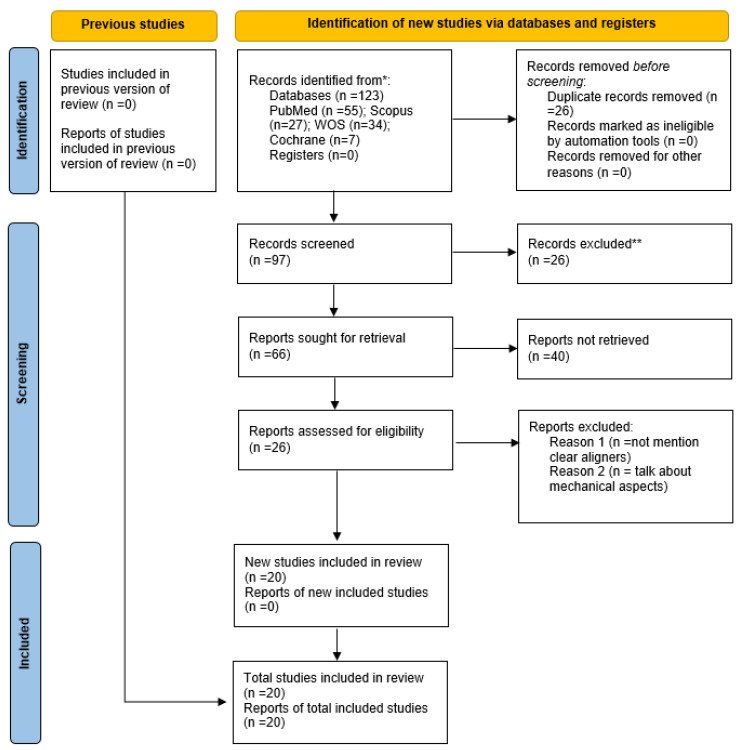
PRISMA flowchart of the systematic review.

Initially, 123 articles were found (55 in PubMed Medline, 7 in Cochrane, 34 in Web of Sciences, and 27 in Scopus). After eliminating duplicates, 98 articles were reviewed, and after reading the title, 67 were selected for abstract reading. Applying the inclusion and exclusion criteria, 27 articles were read in full, and 20 were included in the systematic review (Figure 1). Two authors (A.B.M. and E.P.P.) selected all the trials included in the systematic review by consensus. The data from these articles are summarized in Table 1 [13,14,15,16,17,18,19,20,21,22,23,24,25,26,27,28,29,30,31,32,33]. The type of biofilm analysis is presented in the table to properly evaluate the results. In addition, the number of samples and the type of orthodontics used in each treatment are detailed. The results of the included articles are shown in Table 2.

### 3.2. Subgingival Versus Supragingival Biofilm

The results of the in vivo articles (Table 2) are shown separately from the in vitro studies in Table 3 and Table 4. In the subgingival biofilm (Table 3), significant changes in the orange and green complexes can be observed. *Capnocytophaga* spp. and *Fusobacterium* spp. showed a significant increase in quantity (*p* < 0.005) [13]. Furthermore, the relative abundances of Streptococcus Mutans increased [22]. This might show a possibility of caries and a risk of periodontitis. The clear aligner performed better in the bacterial morphotype analysis than the multi-bracket treatment [18]. One article also observed that only the biofilm was altered in the bracket group, but it did not vary in the clear aligner group [26]. On the other hand, in the supragingival plaque (Table 4), the results were more variable, as were the methodologies used in the articles. Some articles observed that the supragingival microbiome changed during clear aligner treatment [16,19,23,28,29,32]. Some articles observed that the prevalence of periodontopathogens and cariogenic bacteria was higher in braces when analyzing supragingival bacteria [20,24,27]. Most articles did not observe many changes before and after clear aligner treatment [15,21,28,31,32]. Two articles found that the microbiological changes were dependent on oral hygiene status [31,32]. Other articles observed dysbiosis during aligner treatment [16,18,23,29].

### 3.3. Biofilm Changes in Saliva

In salivary analysis, fewer loads of Streptococcus Mutans were obtained with clear aligners compared to when multibracket appliances were used [27]. However, another trial did not obtain the same results [31]. No changes in Lactobacillus were observed with both appliances [27]. Firmicutes and Neissera were also augmented with aligners, but this alteration was only observed in patients with poor oral hygiene (Table 5).

### 3.4. Composition of the Microbiota Before and During Clear Aligner Treatment

During the treatment with clear aligners, changes in the biofilm occurred depending on the type of bacteria [13,14]. Significant changes were observed in the red and orange complexes, especially in *Fusobacterium* spp [13]. Elevated levels of Capnocytophaga, Neisseria, and Arachnia were observed after 3 months of treatment with Invisalign in an in vivo study in adolescents [16]. Wang et al. observed a homogenization of the microbiota in subjects treated with aligners (*p* = 0.0031) compared to untreated subjects, obtaining a microbiota in which 98.7% were from the Firmicutes, Bacteroidetes, Candida division TM7, Proteobacteria, Actinobacteria, Fusobacteria, and Spirochetes families. A total of 124 different genera were found [28]. Before treatment, Zhao et al. observed a microbiota composed of 37% Firmicutes, 21% Proteobacteria, 21% Bacteroidetes, 11% Fusobacteria, 7% Actinobacteria, and1% Candidate division TM7. Both both before and during treatment, the most abundant species were, in order: *Prevotella pallens* ATCC 700821, *Actinomyces graevenitzii* F0530, *Leptotrichia* sp. Oral clone EI013, *Streptococcus parasanguinis* FW213, and *Leptotrichia* sp. Oral clone FP036 14 [31].

Furthermore, Yan et al. obtained a statistically lower Shannon Index after 24 h aligner use (*p* < 0.05) compared to the initial situation and observed an increase in the Simpson Index, without statistical significance. They also observed an increase in beta diversity after 24 h, with a decrease in bacterial density as the hours of aligner use increased [29]. Likewise, on the aligner’s surface, the abundance of some specifically analyzed bacteria gradually decreased with use, showing statistical significance after 12 h of use. Despite this, the amount of Firmicutes increased significantly, from 31% to 56% (*p* < 0.01), as did the Streptococcus genus (*p* < 0.05), 24 h after use of the aligners. On the other hand, the abundance of the Rothia and Actinomyces genera decreased (*p* < 0.05) after 12 h of use. When analyzing the bacterial species, a significant increase in Streptococcus infantis was observed, from 16% at the beginning of treatment to 39% at 24 h, and a significant decrease in *Rothia aeria*, from 3% to 0.34%, was observed 12 h after the start of treatment [27]. Zhao et al. observed similar values of microbiota richness and diversity after 6 months of treatment with aligners (*p* > 0.05), obtaining similar OTU and Shannon–Simpson index values. However, they observed a statistically significant decrease (*p* < 0.05) in the abundance of *Prevotella Pallens* ATCC 700821 after 6 months of treatment with aligners. When periodontal and cariogenic pathogens were specifically evaluated, the relative abundance of Streptococcus mutans and *Streptococcus sobrinus* was 0%, and that of *P. gingivalis* and *T. denticola* was less than 1%, both before and after 6 months of aligner treatment [31]. In addition, the use of computer-aided design and manufacturing (CAD-CAM) materials for clinical aligner fabrication was studied. No differences in biofilm were found among the five different studied materials, but increases in bacterial counts were observed after the third day for all of them [17].

### 3.5. Composition of the Microbiota with Aligners Versus Multibrackets Appliances

Gong et al. conducted an in vivo study in which quantitative and qualitative analyses of the biofilm were performed. In this trial, no significant differences were observed between fixed appliances and clear aligners [29]. Patients with braces obtained worse hygiene indices than patients with clear aligners, while the oral biofilm was the same in terms of its composition [32]. Lombardo et al. found a decrease in TBL with the use of aligners and fixed orthodontics in the first month of treatment, without statistical significance. On the other hand, three months after the start of treatment, a significant increase (*p* = 0.033) was observed in the group with fixed orthodontics, while this increase was not significant in the group using aligners (*p* = 0.084) [28]. Wang et al. observed a significantly higher abundance of Firmicutes (*p* < 0.05) in subjects treated with fixed orthodontics with multibrackets than in untreated patients. When the orthodontic treatment used aligners, a higher abundance was obtained without statistical significance. The proportion of Bacteroidetes was lower in both groups compared to the control group, although only this variation was only significant in the group with multi-bracket fixed orthodontics. Variations were also obtained in the abundance of candidate division TM7, which was significantly higher in both the group treated with aligners and in the group with multi-bracket fixed orthodontics [33]. In the study by Mummolo et al., the number of subjects treated with orthodontics who were observed to have what are considered high-risk values (CFU/mL > 105) of *S. Mutans* and Lactobacillus was evaluated. They observed that, during treatment with aligners, no patient presented with high-risk values in the first 3 months of treatment. However, with multi-bracket appliances, 20% of patients exceeded the risk value at 3 months (*p* = 0.02), and 37.5% at 6 months (*p* = 0.001), for both Lactobacillus and *S. mutans* [27]. Gujar et al. compared the use of aligners with buccal and lingual fixed orthodontics. In their study, there was a statistically significant difference in the amount of *Fusobacterium periodontium* and *Fusobacterium nucleatum*, with high superinfection levels of in 15% and 55% of patients with aligners, levels of 5% and 35% in patients with vestibular fixed orthodontics, and levels of and 5% and 0% in patients with fixed lingual orthodontics, respectively [30]. Sifakakis et al. did not find statistically significant variations (*p* > 0.05) in the relative abundance of *S. Mutans*, *Lactobacillus acidophilus* and *S. Sanguis* between the start of treatment and 1 month after starting treatment with aligners and self-ligating brackets. However, they did find statistical significance when comparing the abundance of these bacteria with that observed in the control group [32].

### 3.6. Periodontal Health

Changes in biofilm composition vary depending on patient characteristics and patient participation. When comparing PIs between patients with aligners and those with multi-bracket fixed orthodontics, Mummolo et al. found statistically higher PIs in patients with braces compared to those who used aligners during the first 6 months. However, these differences were not significant compared to the initial PI values. Sifakakis et al. observed higher PIs and GIs (*p* < 0.05) at 2 weeks and 1 month after fixed orthodontic treatment compared to the aligner group; however, the GI was significantly higher in the fixed-appliance group before the start of treatment. Furthermore, significant differences in oral hygiene were found based on sex: statistically higher initial and treatment-related PIs were obtained in male patients [29,31,32].

### 3.7. Analysis of Biases

Risk of bias was assessed using the JADAD scale (Table 6). The evidence is of low to moderate quality. Most trials were conducted in vitro [11,14,16,18,19,20,21], but some were in vivo trials [12,14,16,34]. Only two of the clinical trials were randomized [12,34], and these were correctly randomized, showing a moderate quality assessment. Non-randomized clinical trials, even when blinded, obtained a low quality assessment, with a score of 2 on the JADAD scale [16,17,19,21,22,23,24,25,27]. Therefore, the quality of the evidence is low to moderate. However, in the last two years, articles have been of higher quality and in vivo studies have been confucted [15,16].

## 4. Discussion

Some authors argue that the microbiota initially tend to homogenize when using transparent aligners [26,27]. Despite this, Firmicutes, Bacteroidetes, Proteobacteria, Actinobacteria and Fusobacteria continued to be predominant in the biofilm, as they were before starting treatment [26,27]. However, an increase in the Firmicutes, Proteobacteria and Actinobacteria families was observed after 6 months of treatment with aligners, whereas the Fusobacteria and Bacteroidetes families decreased with respect to the initial situation. In Wang’s study, the increase in Firmicutes and the decrease in Bacteroidetes were not statistically significant following the use of aligners but they were when using vestibular braces [26]. Yan’s study showed a significant increase in Firmicutes from 31% to 56% (*p* < 0.01) after 24 h of aligner use [28]. When evaluating changes in bacteria considered pathogenic, Zhao et al. observed minimal amounts of *S. mutans*, *S. sobrinus*, *P. gingivalis* and *Treponema denticola* after 6 months of treatment with aligners [26]. In contrast, Mummolo et al. observed that after 6 months of treatment, 8% of patients had values higher than 105 CFU/mL of *S. mutans* and 2.5% of Lactobacillus, although these percentages are much lower than those observed when using multi-bracket vestibular appliances [31]. In another study, a high superinfection of *Fusobacterium periodontum* was observed in 15% of patietns, and *Fusobacterium nucleatum* in 55% of patients with aligners, compared to the rates of 5% and 0% observed when using multi-bracket lingual orthodontics and 5% and 35% when using multi-bracket vestibular orthodontics, respectively [31]. When compared with self-ligating braces, no significant variations were found with respect to the use of aligners in the abundance of *S. mutans*, *Lactobacillus acidophilus*, and *Streptococcus sanguis* [32]. Microbiota analysis allows for an assessment of the health or disease present in any tissue; an association was found between intestinal microbiota dysbiosis and infections, inflammation, cancer, and even autoimmune and degenerative diseases such as Alzheimer’s [33]. The observation of variations in Firmicutes and Bacteroidetes is of particular relevance. Bacteroidetes form a highly heterogeneous family, which is considered beneficial for the body, due to their important metabolic function in ensuring the homeostasis of the intestinal tract mucosa. A decrease in bacteroidetes has been linked to the onset of various oral diseases, such as rheumatoid arthritis or Sjögren’s syndrome. However, an increase in the proportion of Firmicutes is a negative characteristic in biofilm analysis [35]. Dysbiosis of the oral microbiota has been observed, with a reduction in the proportion of Bacteroidetes and an increase in Firmicutes, in cases of oral squamous cell carcinoma [36]. During individual development, dysbiosis occurs in the microbiota, which is dependent on diet. A period of changes in the microbiota occurs during early childhood, beginning with the introduction of solid foods. A second period occurs during adolescence, until a stable composition is reached in adulthood. Diet plays a relevant role in the composition of the microbiota, with a high proportion of Actinomyces and a high proportion of Firmicutes/Bacteroidetes observed in obese adolescents, which, if stabilized, will continuously affect adulthood [37]. Risk factors for periodontal disease include socioeconomic status, the composition and quantity of bacterial plaque, lifestyle, and systemic diseases such as diabetes [38]. If these risk factors were analyzed, it would be observed that they are ultimately all influenced by lifestyle, diet, and oral hygiene habits [39]. Diet is also a key factor in periodontal disease, as a diet deficient in minerals and vitamins from plant foods, such as vitamins A, E, and C has been shown to be highly correlated with the progression of periodontal disease. These have an important antioxidant function, inhibiting the adhesion of pathogens present in plaque and improving cellular immunity. Conversely, a high-carbohydrate diet increases periodontal inflammation. Vitamin C deficiency, both in intake and plasma concentration, increases the prevalence of periodontitis. Vitamin B12, magnesium, and calcium have also been shown to be strongly associated with periodontal progression and destruction [40].

Furthermore, oral hygiene is often a problem during orthodontic treatment, as the plaque index tends to worsen. Oral hygiene monitoring and explanations from dental professionals are essential. The fact that they are removable makes clear aligners an interesting option for adult patients with previous periodontal problems. It seems that the archwire and the bracket promote the adhesion and accumulation of biofilm, making them difficult to remove correctly [41]. However, there is moderate evidence of an association between orthodontic treatment and biofilm dysbiosis [42]. A recent review concluded that orthodontics can lead to dysbiosis, but recommends aligners for the periodontal patient due to the better hygiene that this device allows [43]. These conclusions are based on a previous meta-analysis of cohort studies in which there was no randomization of the subjects, nor was a blinding process was performed. The studies were based on the use of conventional fixed vestibular appliances with multiple brackets [44]. Another meta-analysis supported this view, concluding that removable appliances lead to a lower PI and GI compared to fixed appliances, regardless of the type of appliance used 11. From another perspective, the increased bacterial proliferation in the subgingival sulcus is thought to be associated with periodontal pocket-widening as a result of orthodontic treatment [45]. Preventive treatment to eliminate pathogens before orthodontic treatment has also been recommended [46].

The limitation of the systematic review lies in the low quality of the included studies. The limited evidence in this field is due to the lack of randomization and blinding in existing studies. Furthermore, only four of the included articles collected the subgingival biofilm, and most analyzed supragingival bacteria. However, as a strength of this review, a comprehensive electronic search was performed, including four databases, so all the most recent evidence in this field should be included.

## 5. Conclusion

Clear aligners produce fewer changes in oral biofilm compared to multiple-bracket applications. Furthermore, clear aligners obtained higher values of SM but this appears to depend on the patient’s oral hygiene status.

## Figures and Tables

**Table 1:** Qualitative analysis of articles included in the systematic review.

Author/Year	Funds	Study Type	Sample (n)	Aligners Sample (n Aligners)	Multibrackets Sample (n Multibrackets)	Follow-Up	Analysis
Kredig et al., 2025 [13]	Invisalign^®^ Research Award	Prospective study	50	50	0	1 year	Quantitative analysis
Pasaougly et al., 2025 [14]	Not specified	Compative study	6	6	0	240 days	Quantitative analysis (SM and LA abundance)
Gong et al., 2024 [15]	Shanghai Municipal HealthCommission and the National NaturalScience Foundation of China	n-RCT	20	10	10	6 months	Quantitative and qualitative analysis
Wang et al., 2024 [16]	Guangdong Medical Research Fund	n-RCT	15	15	0	3 months	Qualitative analysis
Moradinezhad et al., 2024 [17]	No funding	In vitro	345	270	0	3 days	Qualitative analysis of selected bacteria
Cenzato et al., 2024 [18]	Partial funding Italian Ministry of Health Current research IRCCS	n-RCT	60	20	20	1 year	Qualitative and semi-quantitative analysis
Zheng et al., 2024 [19]	Beijing Natural Science Foundation, University School and Hospital of Stomatology, Peking University School and Hospital of Stomatology First Clinical Division	n-RCT	48	18	17	nAv	Quantitative and qualitative analysis
Wang et al., 2024 [20]	National Natural Science Foundationof China and Construction EngineeringSpecial Fund ‘Taishan Scholars’	n-RCT	21	10	11	6 months	Qualitative and quantitative analysis (relative abundances)
Cheng et al., 2024 [21]	China Oral Health Foundation, National Natural Science Foundation of China, Nature Science Foundation of Sichuan and Align Technology	n-RCT	25	25	0	6 months	Qualitative analysis
Rouzi et al., 2023 [22]	National Nature Science Foundation of China	n-RCT	15	15	0	3 months	Qualitative and quantitative analysis (abundance of SM)
Song et al., 2023 [23]	National Clinical Research Center for Oral Diseases Key Research and Development Program of Shaanxi Province	n-RCT	55	55	0	nAv	Quantitative analysis (relative abundance of selected bacteria)
Shokeen et al., 2022 [24]	University of Buffalo and The Forsyth Institute	n-RCT	12	6	6	12 months	Qualitative analysis, plaque and gingival index.
Cenzato et. al., 2022 [25]	No funding.	n-RCT	16	8	8	nAv	Quantitative analysis of selected groups of bacteria.
Lombardo et. al., 2021 [26]	No funding.	n-RCT	27	14	13	6 months	Qualitative analysis
Mummolo et al., 2020 [27]	No funding	CC	80	40	40	6 months	Quantitative analysis (SM and LB abundance)
Yan et al., 2020 [28]	National Science Foundation of China.	CH	8	8	0	24 h	Quantitative (relative abundance) and qualitative analysis (alpha and beta diversity, Shannon Index, Simpson Index)
Gujar et al., 2020 [29]	Not specified.	CC	60	20	40	1 month	Quantitative analysis (Relative abundance)
Zhao et al., 2019 [30]	Align Technology. Invisalign^®^	CH	25	25	0	6 months	Quantitative (abundance coverage estimator) and qualitative analysis (alpha and beta diversity, Chaol index, Shannon Index, Simpson Index)
Sifakakis et al., 2018 [31]	No funding	CH	30	15	15	1 month	Qualitative analysis (descriptive PCR)
Wa et al., 2018 [32]	No specified	CC	26	5	5	6 months	Qualitative (OUT) and quantitarive analysis (Shannon Index)

CC: case-control study; CH: cohort study; n-RCT: non randomized clinical trial; SM: Streptococcus Mutans; LB: Lactobacillus; LA: Lactobacillus acidophilus.

**Table 2:** Results of the in vitro studies.

Author	Population	Intervention	Comparison	Outcomes
Pasaougly et al. [14]	In vitro biofilm	Bacterial suspension and optical density of LA and SM on different aligners.	Before treatment; 24 h, 48 h, 72 h, 96 h, 120 h, 168 h, and 240 h after the initiation of treatment; and 1 year post-treatment.	SM and LA formed more biofilm at 120 and 168 h when using Graphy than when using Invisalign (*p* < 0.05).
Moradinezhaz et al. [17]	In vitro specimens	Biofilm analysis using ELISA of microorganisms cultured discs.	Before treatment, and after 24, 72, and 120 h. Comparing plastic materials (polyethylene terephthalate glycol, MMA-free polymer, poly-ethylene, and polyester based on terephthalic acid).	No differences in terms of bacteria growth between plastic materials, but the *Candida albicans* biofilm was more variable in pattern.

**Table 3:** Results of the in vivo studies looking at the subgingival sulculus biofilm.

Author	Population	Intervention	Comparison	Outcomes
Kreding et al. [13]	Fifty adolescent patients (13.3 ± 1.8 years).	Gingival sulcus fluid collected with paper strips. DNA analysis of 11 periodontopathogenic bacteria.	Before treatment. 1 week, 4 weeks, 6 months, 1 year, and 30 months after the start of treatment. One year post-treatment.	Signifcant changes occurred in the orange and green complexes, particularly *Capnocytophaga* spp. (*p* = 0.0042) and *Fusobacterium* spp. (*p* = 0.0365).
Cenzato et al. [18]	Sixty patients (12–65 years old).	Bacterial morphotype analysis of subgingival analysis.	Control group vs. clear aligners vs. fixed bracket appliances.	Clear aligners showed better periodontal bacteria status than conventional brackets.
Rouzi et al. [22]	Fifteen patients (19–35 years old)	Subgingival sulculus and plaque from the inner surface of the aligner analyzed via 16S rRNA gene sequencing.	Before treatment, and after 1 and 3 months.	The relative abundance of SM increased significantly during aligner treatment, but the alpha and beta diversity were similar.
Lombardo et al. [26]	Twenty-seven patients (13–22 years old)	Subgingival fluid PCR	Fixed appliances vs. clear aligners. Before treatment, 3 and 6 months of treatment.	Total bacterial load did not vary in the clear aligner group, but it did in the bracket group.

SM: Streptococcus mutans, LA: lactobacillus acidophilus, PCR: polymerase chain reaction, rRNA: ribosomal ribonucleic acid, DNA: desoxirribonucleic acid.

**Table 4:** Results of the in vivo trials collected from supragingival plaque.

Author	Population	Intervention	Comparison	Outcomes
Gong et al. [15]	Patients between 11 and 34 years old.	Supragingival plaques analyzed by 16S rRNA gene sequiencing and chromatography.	Fixed multibracket appliances vs. clear aligners. Before treatment, after 1–3 months and after 6 months of treatment.	No significant changes were observed in alpha and beta diversity but the relative abundace of Veillonella, Mogibacterium and Actinomyces experienced the most significant changes.
Wang et al. [16]	Fifteen adolescents (12–15 yearsold).	Supragingival plaque DNA extraction and metagenomic sequencing.	Before tratment and 3 months of treatment.	Higher relative abundance of Capnocytophaga, Neisseria, and Arachnia after 3 months of treatment. Also the virulence factor associated with type IV pili was higher than before treatment.
Zheng et al. [19]	Forty eight female patients (18–38 years old)	16S rRNA gene sequencing of selected bacteria.	Control vs. bracket vs. aligner. After eating and after brushing teeth.	Actinobacteriota was significantly more prevalent in the control group, Lautropia in the aligner group and Prevotellacae in the brackets group.
Wang et al. [20]	Twenty-one patients (11–30 years old)	16S rDNA gene PCR.	Bracket vs. clear aligner group. Before treatment, three months and six months of treatment.	Beta diversity showed different biofilm in the aligners and bracket group. Periodontopathogens might be more present in the bracket group.
Cheng et al. [21]	Twenty five children	Supragingival 16S rDNA gene sequencing	Bedore treatment and after 6 months.	No statistical differences were found between groups and time points (*p* > 0.05)
Shokeen et al. [24]	Twelve patients (8–56 years old)	Supragingival plaque analysis with 16S rRNA gene sequencing.	Fixed bracket vs. clear aligners. After 1, 3, 6 and 12 months of treatment.	Only beta but not alpha communities were diferent between aligner and bracket treatment. Aligner showed better microbial outcomes.
Cenzato et al. [25]	Sixteen patients (7–35 years old)	Supragingival plaque sample from lower right first molars gene gram staining procedures.	Fixed appliances vs. clear aligners.	The bracket group showed 25% less gram+ cocci, 50% more Gram− cocci, 40% more gram+ bacilli and 12% more gram− bacilli than aligners group.
Yan et al. [28]	Eight female patients (18–25 years)	Inner aligner surface plaque analysis with 16rRNA gene sequencing	Before treatment, after 4, 8, 12 and 24 h.	The beta diversity did not suffer significant increases after 24 months of treatment with aligners. The relative abundances of Firmicutes and Bacteroidales increased while Actinomyces and Rothia decreased.
Gujar et al. [29]	Sixty patients	Plaque of the appliance surface with DNA-DNA hybridization.	Clear aligner vs. labial bracket vs. lingual bracket after 30 days of use	Treponema denticola statistically increased in all the appliances.
Zhao et al. [30]	Twenty five adult patients	16S rRNA gene sequiencing	Before and after 6 months of clear aligner treatment (Invisalign).	The biofilm did not change in patients with good oral hygiene and aligners.

SM: *Streptococcus mutans*, LA: *Lactobacillus acidophilus*, PCR: Polymerase chain reaction, rRNA: Ribosomal ribonucleic acid, DNA: Desoxirribonucleic acid.

**Table 5:** Results of the in vivo trials collected from saliva.

Authors	Population	Intervention	Comparison	Outcomes
Song et al. [23]	Fifty five patients (11–18 years old)	Saliva 16S rRNA gene sequencing and chromatography mass spectrometry.	Control group vs clear aligners after 3 months.	Lachnoanaerobaculum, Rothia, Subdoligranulum and some aminoacids were increased during aligner treatment.
Mummolo et al. [27]	Eighty patients (19–24 years old)	Salivary levels of SM and Lactobacillus.	Before treatment, after 3 and 6 months of treatment.	Lower colonizations of SM and Lactobacillus was observed with aligners compared to bracket appliances.
Sifikakis et al. [31]	Thirty adolescent patients	Salivary bacterial analysis with PCR	Self-ligating bracket with nickel-titanium archwire vs aligner of polyethylenterephthalat-glycol copolyester. Before, after 2 weeks and 1 month.	Even participants in the aligner group have better hygiene than in the bracket group, no higher loads of SM were observed.
Wang et al. [32]	Twenty six patients (20–25 years)	Saliva 16S rRNA analysis	Control vs fixed appliances vs clear aligner (Invisalign)	Firmicutes and Neisseria showed significant differences. Aligners treatment showed disbyiosis being dependent of patient hygiene.

SM: Streptococcus mutans, PCR: Polymerase chain reaction, rRNA: Ribosomal ribonucleic acid.

**Table 6:** Assessment of methodological quality according to the Jadad scale.

JADAD CRITERIA						
Author/Year	Is the Study Described as Randomized?	Is the Study Described as Double-Blinded?	Was There a Description of Withdrawals and Dropouts?	Was the Method of Randomization Adequate?	Was the Method of Blinding Appropriate?	Score
Kredig et al., 2025 [13]	0	NA	NA	0	NA	NA
Pasaougly et al., 2025 [14]	0	NA	NA	0	NA	NA
Gong et al., 2024 [15]	0	1	1	0	0	2
Wang et al., 2024 [16]	0	1	1	0	0	2
Moradinezhard et al., 2024 [17]	0	NA	1	NA	NA	1
Cenzato et al., 2024 [18]	0	1	1	0	0	2
Zheng et al., 2024 [19]	0	1	0	NA	0	1
Wang et al., 2024 [20]	0	1	1	0	0	2
Cheng et al., 2024 [21]	0	1	1	0	0	2
Rouzi et al., 2023 [22]	0	1	1	0	0	2
Soung et al., [23]	0	1	1	0	0	2
Shokeem et al., 2022 [24]	0	1	1	0	0	2
Cenzato et al., 2022 [25]	0	0	1	0	0	1
Lombardo et al., 2021 [26]	0	0	1	0	0	1
Mummolo et al., 2020 [27]	0	NA	0	NA	NA	NA
Yan et al., 2020 [28]	0	NA	NA	0	NA	NA
Gujar et al., 2020 [29]	0	NA	NA	0	NA	NA
Zhao et al., 2019 [30]	0	NA	NA	0	NA	NA
Sifakakis et al., 2018 [31]	0	NA	NA	0	NA	NA
Wang et al., 2018 [32]	0	NA	NA	0	NA	NA

NA: Not applicable.

## Data Availability

The original contributions presented in this study are included in the article. Further inquiries can be directed to the corresponding author.
